# CodeStream: A dataset of iterative programming submissions with sequential verdict traces and attempt histories

**DOI:** 10.1016/j.dib.2026.112843

**Published:** 2026-05-12

**Authors:** Nazira Jesmin Lina, Syed Mumtahin Mahmud, Mahmudul Hasan, Md Fahim Arefin, Redwan Ahmed Rizvee, Md Mahmudur Rahman, Md Mosaddek Khan

**Affiliations:** Department of Computer Science and Engineering, University of Dhaka, Dhaka 1000, Bangladesh

**Keywords:** Automated code assessment, Submission behavior analysis, Computer science education, Coding practice platforms, Educational Data Mining

## Abstract

Programming learning environments generate rich interaction data through iterative code submissions and automated evaluation processes. This article presents CodeStream, a dataset of programming submissions collected from undergraduate computer science students during supervised problem-solving sessions using an automated assessment platform. The dataset contains 5482 submissions from 202 participants across 46 programming problems written in C, C++, and Java. Each submission record includes source code, programming language, final evaluation verdict, attempt order, and sequential verdict traces generated during test case evaluation. A linked problem-level component provides problem descriptions and associated evaluation test cases. The dataset preserves temporal relationships between users, problems, and attempts, enabling reconstruction of submission histories and analysis of iterative problem-solving behavior. CodeStream supports research in educational data mining, learning analytics, automated feedback systems, code analysis, and programming behavior modeling. Its attempt-level structure is particularly suitable for studying error correction patterns, learning progression, and sequential decision-making in novice programming contexts.

Specifications Table

**Table:** 

Subject	Computer Science
Specific subject area	Programming learning analytics using iterative code submissions and automated evaluation
Type of data	Table, CSV files.
Data collection	Data were gathered from proctored programming sessions in which undergraduate CS students took part on a voluntary and informed-consent basis. Participants solved problems independently on a custom assessment platform, with internet access restricted to a single whitelisted resource and screen-switching prohibited, reducing the likelihood of external assistance. The platform enforced uniform execution constraints (3-second time limit, 256 MB memory cap) and automatically assigned verdicts upon each submission. All submission events, including attempt order, language choice, source code, and verdict outcomes, were captured directly from platform logs without manual intervention.
Data source location	Department of Computer Science and Engineering, University of Dhaka, Dhaka - 1000, Bangladesh 23°43′42.0″N 90°23′54.6″E
Data accessibility	Repository name: CodeStream: A Dataset of Iterative Programming Submissions with Sequential Verdict Traces and Attempt Histories Data identification number: 10.17632/n77t7z9zcr.1 Direct URL to data: https://data.mendeley.com/datasets/n77t7z9zcr/1
Related research article	None

## 1 Value of the Data

• The dataset offers significant value for studying learning dynamics in novice programmers in undergraduate CS courses. Submission attempt numbers and sequential verdict histories let researchers analyze iterative problem-solving behavior, error-correction behaviors, and iterative problem-solving patterns. This makes the data particularly useful for educational data mining and learning analytics, where understanding how users improve across attempts is essential.
• Having both final verdicts and complete verdict sequences provides a unique opportunity to investigate evaluation systems and feedback mechanisms. Researchers can examine how intermediate outcomes (e.g., compilation errors, wrong answers) evolve into final accepted solutions, enabling the development of automated feedback systems and intelligent tutoring models.
• The dataset supports research in software engineering and code analysis. Raw source code strings linked to outcomes let researchers explore code quality, stylistic patterns, and language-specific performance. This can facilitate advances in code recommendation systems, automated grading, and bug-detection algorithms.
• The dataset enables behavioral analysis of user interaction patterns through submission frequency, programming language usage, and success rates. While individual learner background information was not recorded to preserve anonymity, all participants were drawn from 1st or 2nd-year undergraduate computer science programs. This provides a consistent academic context for interpreting learning trajectories and engagement patterns, while maintaining ethical data collection standards.

## 2 Background

The integration of automated assessment systems in computer science education has reshaped how programming skills are evaluated and practiced. Online judge platforms execute submitted code against predefined test cases, returning structured verdicts (Accepted, Wrong Answer, Runtime Error, and similar categories) that enable scalable, objective evaluation across large cohorts. These pipelines, grounded in test-driven evaluation methodologies, have been examined in foundational reviews of automated programming assessment [1,2] and in more recent surveys of online judge systems and their use in instruction [3].

From a pedagogical perspective, such environments align with constructive learning theories in which iterative cycles of submission, feedback, and refinement support skill acquisition. The fine-grained interaction data produced by these systems has driven the growth of educational data mining and learning analytics, where attempt sequences are used to model learner behavior, predict outcomes, and design adaptive feedback [4].

Several large-scale datasets have been released to support this line of work. BlueJ Blackbox captures IDE-level activity, including compilations and edits, from novice Java programmers worldwide [5]. CodeWorkout, released through the CSEDM Data Challenge, provides CS1 Java submission traces with attempt sequences from a US public university [6]. CodeNet aggregates over 14 million judged submissions across 55 languages drawn from competitive programming platforms [7]. The ProgSnap2 specification has emerged as a community standard for representing programming process data across such collections, supporting cross-dataset analyses [8].

CodeStream complements these resources along three dimensions. It captures attempt-ordered submissions with full verdict sequence traces (rather than only final outcomes), is collected under proctored conditions that ensure the data reflects unaided problem-solving, and draws from a multi-language (C, C++, Java) cohort at a South Asian institution, a population underrepresented in existing public datasets.

## 3 Data Description

The dataset [9] provides a structured record of how users iteratively solve programming problems, capturing both problem definitions and detailed submission records associated with user interactions. It organizes data at multiple levels of granularity, including individual submissions, sequential attempts, and problem-specific test cases. The dataset integrates identifiers, source code, outcome verdicts, and sequential evaluation traces, allowing consistent linkage across its components. This structure helps the researchers easily explore the data and understand both submission details and problem details in a connected way.

### 3.1 Repository structure

The dataset is organized in a repository structure comprising two primary data components and an accompanying documentation file. The structure is designed to facilitate accessibility, reproducibility, and clear separation between submission-level records and problem-level definitions.

The two data files described in Table 1 are connected via a shared *problem_id*, enabling relational mapping between submissions and their corresponding problem specifications. In addition to the primary data file, the repository includes supplementary documentation that describes column definitions, the data schema, and usage guidelines. These materials support reproducibility and facilitate the reuse of the dataset across different research contexts.

**Table 1:** Overview of the primary data files included in CodeStream and their corresponding descriptions.

File Name	Description
Submission Data.csv	Records of individual code submissions across users and attempts
Problem Data.csv	Definitions of programming problems and their test cases

#### 3.1.1 Overall dataset composition

The dataset contains:

• 5482 submission records
• 46 unique programming problems
• 202 unique users

Each submission represents a single attempt made by a user for a specific problem, forming a one-to-many relationship from both users and problems to submissions.

### 3.2 Submission data

The submission-level component captures detailed records of user interactions with programming problems. Each entry corresponds to a single submission instance and documents how the participant attempts progress over time.

#### 3.2.1 Variables

Table 2 summarizes each variable in the submission-level dataset, describing what it records and how it contributes to representing user submissions and automated evaluation records. Together, these fields enable reconstruction of a user's full problem-solving trajectory, linking each submission to its author, the target problem, and the evaluation results it received.

**Table 2:** Description of variables contained in the submission-level dataset, including identifiers, attempt information, source code, and evaluation outcomes.

Variable Name	Description
*submission_id*	Unique identifier for each submission
*user_id*	Identifier representing an individual user
*problem_id*	An identifier linking the submission to a specific problem
*attempt_number*	Sequential index of attempts for a given user–problem pair
*programming_language*	Programming language used in the submission
*source_code*	Full source code submitted by the user
*final_verdict*	Final outcome assigned after evaluation
*verdict_sequence_trace*	Ordered sequence of evaluation results during execution

#### 3.2.2 Structural characteristics

• Multiple submissions are associated with the same *(user_id, problem_id)* pair, forming attempt sequences.
• The *attempt_number* variable represents the temporal order of submissions.
• The verdict_sequence_trace captures the progression of evaluation outcomes within a submission. Specifically, for each problem’s evaluation_test_cases, the trace encodes the outcome of individual test cases in their execution order. For example, a verdict_sequence_trace ["Wrong Answer", "Wrong Answer", "Accepted"] indicates the solution failed on the first two test cases and produced the correct result on the last one.

#### 3.2.3 Programming language distribution

Each submission is associated with a single programming language, allowing categorization by language choice. The dataset includes submissions written in three primary programming languages, with additional statistics and structural summaries presented in Table 3, Table 4, Table 5, Table 6, Table 7.

**Table 3:** Distribution of programming submissions across the supported programming languages in the dataset.

Language	Number of Submissions
C	2112
Java	2086
C++	1284

**Table 4:** Frequency distribution of automated evaluation verdicts assigned to programming submissions.

Verdict Type	Frequency
Wrong Answer	1656
Accepted	1649
Compilation Error	1043
Presentation Error	732
Runtime Error	339
Time Limit Exceeded	62
Memory Limit Exceeded	1

**Table 5:** Description of variables included in the problem-level dataset.

Variable Name	Description
*problem_id*	Unique identifier for each problem
*problem_description*	Textual specification describing the problem
*evaluation_test_cases*	Structured representation of input–output test cases

**Table 6:** Categorization of programming problems according to their primary algorithmic or computational topic.

Primary Topic	Problem IDs
String Processing	prob_001, prob_002, prob_003, prob_005, prob_011, prob_017, prob_023, prob_030, prob_035, prob_043
Array / Data Structures	prob_006, prob_008, prob_013, prob_018, prob_022, prob_027, prob_031, prob_037, prob_041, prob_045
Mathematics / Number Theory	prob_007, prob_009, prob_012, prob_016, prob_020, prob_024, prob_028, prob_032, prob_036, prob_040, prob_044
Graph Algorithms	prob_015, prob_026, prob_039
Dynamic Programming / Sequences	prob_021, prob_034
Greedy Algorithms	prob_004, prob_010, prob_019, prob_025, prob_029, prob_033
Sorting and Searching	prob_014, prob_038, prob_042, prob_046

**Table 7:** Summary of the major dataset components, including units of observation, record counts, and key identifiers.

Component	Unit of Observation	Number of Records	Key Identifier
Submission Data	Individual submission	5482	submission_id
Problem Data	Programming problem	46	problem_id

#### 3.2.4 Final verdict distribution

The evaluation outcomes associated with submission records are categorized into distinct verdict types assigned by the automated assessment system after code compilation and execution. Each submission is evaluated against predefined test cases, and the resulting verdict reflects the correctness, performance, or execution status of the submitted program. The distribution presented below summarizes the frequency of these verdict categories across all submissions in the dataset.(Tables 4, 5, 6)

**Fig. 1:**
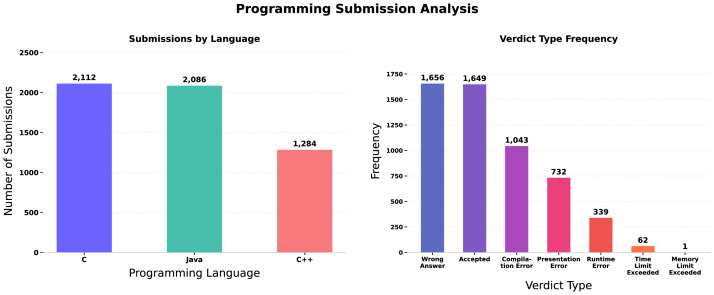
Distribution of submissions by programming language and evaluation verdict type.

Fig. 1 indicates that C and Java are the most frequently used languages with comparable submission volumes, while Wrong Answer and Accepted constitute the majority of verdict outcomes.

### 3.3 Problem data

The problem-level component represents the static aspect of the dataset, providing definitions of programming tasks and their associated test cases. Each record corresponds to a distinct problem and includes the information needed to understand the problem statement and its evaluation mechanism. Submission records are linked to this component through a shared problem ID, allowing each submission to be interpreted within the context of its target problem.

#### 3.3.1 Variables

The problem dataset includes variables that describe the identity and specification of each programming problem. These variables comprise unique identifiers, textual problem descriptions, and structured representations of evaluation test cases, which collectively define the input–output conditions used during automated assessment.

#### 3.3.2 Structural characteristics

• The dataset includes 46 distinct problems, each uniquely identified.
• Each problem contains a textual description and associated evaluation criteria in the form of test cases.
• The evaluation test cases are stored as JSON objects.

### 3.4 Problem categorization

The problems in the dataset are categorized based on their dominant algorithmic or programming concept, as inferred from their problem structure and expected solution approach. Each problem is assigned to a specific topic (such as string processing, array and data structures, graph algorithms, dynamic programming, greedy methods, or mathematical reasoning) reflecting the primary technique required for its solution. This approach avoids overly generic classifications and ensures that each problem is mapped to a clearly defined computational paradigm, enabling more meaningful analysis of problem-solving patterns and student performance across different algorithmic domains.

### 3.5 Data linkage and relational structure

**Fig. 2:**
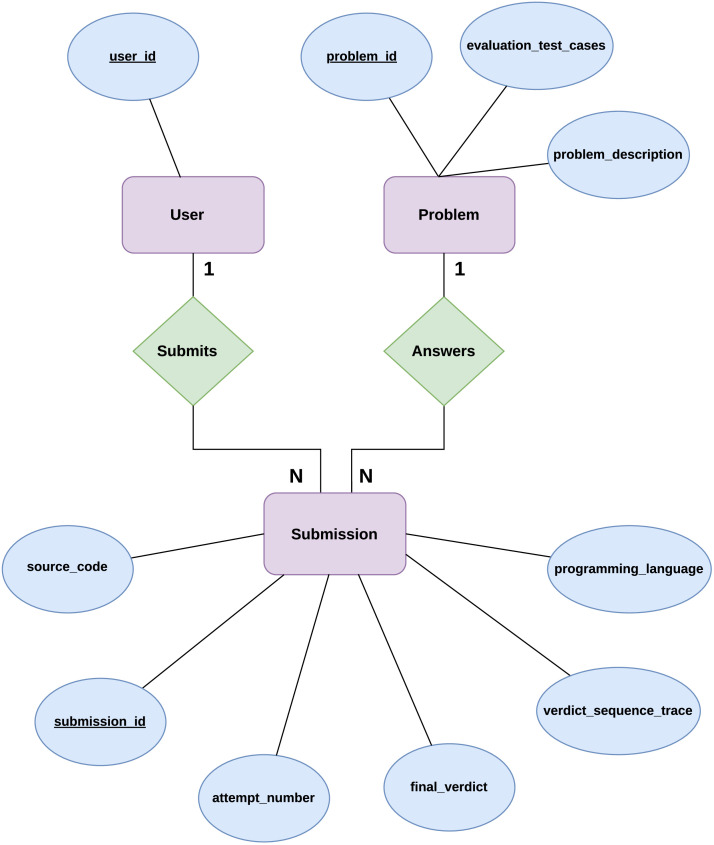
Entity–relationship schema of the dataset.

The relationships among users, submissions, and problems are illustrated in Fig. 2, which presents the entity–relationship schema of the dataset. The diagram shows that each submission is linked to a single user and a single problem through foreign key relationships, forming a one-to-many association from both users and problems to submissions. It also depicts the submission entity as a central table that stores attempt-level attributes, including programming language, verdict outcomes, and source code. The dataset follows a relational structure centered on the *problem_id*:

• Each submission record references exactly one problem.
• Each problem may be associated with multiple submissions from different users.
• Users may submit solutions to multiple problems, and multiple times per problem.

This structure enables the reconstruction of:

• Submission histories per user
• Attempt sequences per problem
• Cross-user interaction patterns with the same problem

## 4 Experimental Design, Materials and Methods

The dataset was collected in a controlled environment using an automated submission and assessment platform, where participation was voluntary. The experimental setup was designed to capture authentic student programming behavior, ensuring that all recorded submissions were independently produced without external assistance.

### 4.1 Data acquisition environment

Data collection took place during structured evaluation sessions, where participants voluntarily engaged in solving predefined programming problems using the designated platform. The environment enforced strict constraints to maintain data integrity. Participants ensured that they did not switch screens, access the internet, or use any external resources during the session. As a result, the submissions are expected to predominantly reflect unaided human problem-solving.

### 4.2 System environment and execution pipeline

All submissions were processed by an automated evaluation system. Each submitted program was subjected to a standardized pipeline consisting of compilation (when required), execution in a controlled runtime environment, and validation against predefined input–output test cases associated with each problem.

The execution environment enforced predefined constraints, including a uniform limit of 3 s for execution time and 256 MB for memory usage across all problems. Evaluation results were generated entirely by the system, without any manual intervention.

### 4.3 Logging mechanism and data capture

The dataset was constructed from system-generated logs that capture all submission-related events. For each submission, the system recorded:

• A unique submission identifier and associated user and problem identifiers
• The chronological order of submissions
• Execution feedback produced during evaluation
• The final evaluation verdict assigned by the system

These logs preserve the full sequence of attempts made by each user for a given problem.

In addition to submission-level logs, problem-level records were retrieved from the platform’s repository. These include problem descriptions and the corresponding evaluation test cases used during automated assessment.

## Limitations

The dataset has several limitations that should be considered by future users. First, the data were collected at a single institution (Department of Computer Science and Engineering, University of Dhaka) from a self-selected pool of voluntary participants. The user population is therefore not representative of novice programmers globally, and findings derived from the dataset may not generalize to other institutional or cultural contexts.

Second, the problem set comprises 46 programming problems, which is modest in scale relative to larger published datasets such as CodeWorkout and CodeNet. The problem set is not stratified by difficulty level, topic, or curricular alignment, and users intending to study learning trajectories should be aware that problem-level variability has not been formally controlled.

Third, the dataset captures three programming languages (C, C++, and Java).

Fourth, contextual information about participants, including academic year, prior programming experience, course enrolment, and session duration, was not recorded in order to preserve anonymity. This restricts the dataset's use for analyses that depend on learner background or temporal session structure.

## Ethics Statement

Participation in the data collection was voluntary, and informed consent was obtained from all participants prior to the session. Participants were briefed on the purpose of the data collection, the categories of data to be recorded (programming submissions, attempt order, and automated evaluation outcomes), the intended research use of the data, and their right to withdraw at any time without consequence.

No personally identifiable information was collected at any stage. User identities were replaced with system-generated identifiers prior to any storage or analysis, and the published dataset contains no names, contact details, demographic attributes, or institutional identifiers linked to individual records. Source code submissions were reviewed prior to release to ensure that no identifying information (such as names or identifiers embedded in comments or string literals) was retained.

The data collection involved adult participants engaged in a routine programming activity that posed no greater risk than ordinary educational practice. The authors confirm that the work complies with the ethical standards of the host institution, as no formal IRB review was required for non-interventional, anonymized educational records.

## CRediT authorship contribution statement

**Nazira Jesmin Lina:** Data curation, Formal analysis, Investigation, Methodology, Visualization, Writing – original draft. **Syed Mumtahin Mahmud:** Data curation, Formal analysis, Investigation, Methodology, Writing – original draft. **Mahmudul Hasan:** Data curation, Funding acquisition, Software. **Md Fahim Arefin:** Conceptualization, Methodology, Project administration, Validation, Writing – review & editing. **Redwan Ahmed Rizvee:** Data curation. **Md Mahmudur Rahman:** Resources. **Md Mosaddek Khan:** Supervision.

## Data Availability

Mendeley DataCodeStream: A Dataset of Iterative Programming Submissions with Sequential Verdict Traces and Attempt Histories (Original data)
